# Impact of a brief educational intervention on eating habits in a sample of Peruvian adolescents aged 10–12 years: a preliminary study

**DOI:** 10.3389/fpubh.2025.1702418

**Published:** 2025-11-10

**Authors:** Yasti Garcia-Castillo, Ariana Elera-Campos, Mery Rodríguez-Vásquez, David Javier-Aliaga

**Affiliations:** Faculty of Health Sciences, School of Nutrition, Universidad Peruana Unión, Lima, Peru

**Keywords:** nutritional education, eating habits, adolescents, nutritional intervention, Peru

## Abstract

**Introduction:**

Currently, the prevalence of overweight and obesity among adolescents is a relevant public health concern. In this context, schools are considered a suitable setting for promoting healthy eating habits from an early age. Within this framework, the present study aimed to preliminarily explore the impact of a brief nutritional education intervention on eating habits in a sample of adolescents aged 10–12 years from a public school in Lima, Peru.

**Materials and methods:**

A quantitative study with a quasi-experimental design was conducted, including an experimental group (*n* = 16) and a control group (*n* = 8), under a pretest–posttest scheme. The final sample consisted of 24 adolescents aged 10–12 years, selected through simple random sampling. An eating habits questionnaire was administered before and after the educational intervention. The educational program lasted 2 months and comprised six sessions.

**Results:**

The brief nutritional program produced a significant improvement in the eating habits of the experimental group (*p* < 0.001), whereas the control group showed no changes (*p* = 0.495). In addition, both groups were equivalent at pretest (*p* = 0.928), but at posttest the experimental group exhibited a significant difference compared to the control group (*p* = 0.004), confirming the effectiveness of the intervention.

**Conclusion:**

The findings of this study provide preliminary evidence that brief educational interventions in school settings may contribute to the improvement of eating habits. These results support the relevance of considering nutritional education as a preventive strategy within public health policies to promote healthy lifestyles from early ages.

## Introduction

Adolescents worldwide are experiencing a critical health crisis due to the increasing prevalence of overweight and obesity. According to the World Health Organization (WHO), obesity affected approximately 160 million young people aged 5–19 years, a condition that was part of a total of 390 million cases of overweight within the same age group (1). Furthermore, a recent meta-analysis including more than 45 million children and adolescents from 154 countries estimated a global obesity prevalence of 8.5% (95% CI: 8.2–8.8), with significant regional variations ranging from 0.4% in Vanuatu to 28.4% in Puerto Rico. In addition, overweight reached 14.8% and excess weight 22.2% in this age group, showing strong associations with risks such as depression and hypertension (2). Other studies have also reported that overweight and obesity increase the risk of developing chronic diseases such as type 2 diabetes, hypertension, dyslipidemia, and metabolic syndrome from early ages. Similarly, evidence shows that these conditions are associated with higher levels of depression, anxiety, and low self-esteem among adolescents (3–5). These consequences reflect that youth obesity constitutes a major public health problem with both physical and emotional implications requiring urgent attention.

In the scientific literature, eating habits or lifestyles related to nutrition are recognized as key determinants of overweight and obesity in adolescents (6–9). In response to this issue, numerous long-term nutritional interventions have been developed. For example, Alemán-Castillo et al. (10) implemented a nutritional education program with a two-year follow-up in schoolchildren aged 8 to 11 years, achieving a significant reduction in calorie, carbohydrate, and sugar intake. Similarly, González et al. (11) applied a Nutritional Education Program (PREN) in children aged 6 to 9 years over the same period, observing a decrease in household energy consumption. Likewise, Arenaza et al. (12) worked with overweight and obese children aged 8 to 12 years through a 22-week family-based healthy lifestyle program, which resulted in significant improvements in diet quality and other health indicators. Complementarily, shorter-term programs—typically lasting between 5 and 12 weeks—have also been implemented. Although still limited in number, these interventions have shown immediate positive effects on eating habits (8, 13–15). For instance, Ahmed et al. (16) conducted a 12-week school-based intervention with adolescents aged 13–17 years, which increased fruit and vegetable consumption while reducing the intake of sugar-sweetened beverages. In this context, there is a need to design and implement brief programs that are culturally adapted to the local environment, fostering immediate changes in schoolchildren’s eating and lifestyle behaviors, with the potential for replicability and scalability as effective strategies for preventing overweight and obesity during adolescence.

In the Peruvian context, the situation of adolescents is equally concerning. According to the most recent UNICEF report, 24.8% of adolescents aged 12–17 years are overweight or obese, while in the group aged 6–13 years the prevalence reaches 38.4% (17). At the national level, the 2024 Demographic and Family Health Survey (ENDES) reported that 62% of Peruvians over 15 years of age are overweight or obese (18). If this trend continues, the combined costs attributable to overweight and obesity in children and adolescents are projected to reach 210.6 billion USD between 2025 and 2092 (19). Despite this critical reality, national research on nutritional educational interventions remains limited, although the available studies have shown encouraging results. For instance, López et al. (14) implemented a 10-week program in schoolchildren aged 5–13 years living under vulnerable conditions, achieving significant improvements in fruit and vegetable consumption, as well as in hygiene and self-care practices. Similarly, Vasquez-Mamani et al. (20) demonstrated significant improvements among adolescents aged 12 to 18 years in Lima after a 16-week intervention, increasing levels of knowledge, attitudes, and dietary practices. Furthermore, in a complementary study conducted in a rural community of Iquitos (Peru), Cusquisibán-Alcantara et al. (21) reported significant improvements in nutritional knowledge, the healthy eating index, and other indicators, also following a 16-week educational intervention. In summary, these findings confirm the relevance of implementing brief and targeted nutritional educational programs, specifically aimed at Peruvian schoolchildren and adolescents, as a preventive strategy against the sustained increase of overweight and obesity in the country.

This research is justified in different areas that support its relevance. In the academic and scientific field, it seeks to provide updated empirical evidence on the effectiveness of brief educational programs aimed at promoting healthy lifestyles among adolescents. Since most interventions described in the international literature are long-term, it is necessary to explore short-term strategies that, with a rigorous design, generate immediate changes in eating habits and are feasible to replicate in resource-limited contexts. In this way, the study helps to fill a gap in research on school nutrition and public health. In the social and health domain, the pertinence of this work is based on the urgency of addressing a current problem that threatens to increase the burden of non-communicable chronic diseases from early ages. The implementation of brief and targeted educational programs therefore constitutes a strategic opportunity to influence the behavioral determinants of obesity and to foster, during adolescence, a culture of self-care that is sustainable throughout life. Finally, in the economic and political sphere, this research offers a cost-effective alternative with the potential to be replicated and scaled within the Peruvian educational system. Given the growing economic impact of excess weight, it is essential to generate sustainable preventive solutions that contribute to reducing health and social costs, while also providing practical inputs for the formulation of comprehensive public health and education policies.

In this context, the present study aimed to preliminarily explore the impact of a brief nutritional education intervention on eating habits in a sample of adolescents aged 10–12 years from a public school in Lima, Peru.

## Materials and methods

### Methodological design

This study was framed within a quantitative approach and employed a quasi-experimental design, involving an experimental group and a control group. The experimental group received the intervention of a nutritional educational program aimed at modifying and improving eating habits, while the control group did not receive any intervention. The study also had a longitudinal component, as it included two measurements: one prior to the intervention (pretest) and another after the intervention (posttest). Finally, the independent variable was the nutritional educational program, and the dependent variable corresponded to the eating habits of the participants (22).

### Sampling design

The population consisted of 50 students from a public educational institution located in Lima, Peru. The initial selection was carried out using a non-probabilistic purposive sampling approach, including only students who met the predefined eligibility criteria. Subsequently, participants were allocated to the experimental and control groups through simple randomization using SPSS statistical software (version 25) via the “Random sample of cases” function. This procedure ensured an impartial allocation, free from biases related to researcher discretion or participant availability. As a result of the randomization process, 16 students were assigned to the experimental group and 8 to the control group. Inclusion criteria were as follows: students of both sexes, aged 10–12 years, of Peruvian nationality, enrolled during the study period, and with informed consent voluntarily signed by a parent or legal guardian. Exclusion criteria included acute infections, food allergies, communication difficulties, or any medical condition that could interfere with changes in eating habits.

### Sociodemographic characteristics of the participants

Table 1 presents the sociodemographic characteristics of adolescents and their mothers in both experimental and control groups. Regarding the sex of the adolescents, females were predominant in both groups (56.3% in the experimental group and 62.5% in the control group). With respect to age, most participants were between 10 and 11 years old (93.8% in the experimental group and 100% in the control group), while the proportion of 12-year-old adolescents was small (6.3%) and observed only in the experimental group. Concerning the mothers, the most frequent age group was 26–35 years (93.8% in the experimental group and 87.5% in the control group), while a smaller proportion corresponded to mothers aged 16–25 years (6.3 and 12.5%, respectively). In terms of educational level, basic education was predominant in both groups (75.0% in the experimental and 62.5% in the control group), although a relevant proportion of mothers had higher education (25.0 and 37.5%, respectively). Regarding geographic origin, mothers in the experimental group came equally from the Coast and the Jungle (37.5% each), while in the control group the Coast predominated (50%), followed by the Jungle and the Highlands (25% each). Finally, in terms of occupation, in the experimental group mothers were distributed similarly between dependent employment (37.5%) and housework (37.5%), with a smaller proportion in independent work (25.0%). In contrast, in the control group, independent work predominated (50%), followed by housework (37.5%) and dependent employment (12.5%).

**Table 1:** Sociodemographic characteristics of mother and child in experimental and control groups.

Characteristics	Experimental group (*n* = 16)		Control group (*n* = 8)	
Child’s characteristics	*n*	%	*n*	%
Sex				
Female	9	56.3	5	62.5
Male	7	43.8	3	37.5
Age				
10 years	9	56.3	4	50
11 years	6	37.5	4	50
12 years	1	6.3	0	0
Mother’s characteristics				
Age				
16–25 years	1	6.3	1	12.5
26–35 years	15	93.8	7	87.5
Educational level				
Basic education	12	75.0	5	62.5
Higher education	4	25.0	3	37.5
Geographic origin				
Coast	6	37.5	4	50
Highlands	4	25.0	2	25
Jungle	6	37.5	2	25
Occupation				
Formal employment	6	37.5	1	12.5
Informal employment	4	25.0	4	50
Housewife	6	37.5	3	37.5

### Variable and instrument

Eating habits were measured using the Eating Habits Questionnaire for Peruvian Adolescents. This instrument was originally developed by Aymar (23) and later adapted by Carrasco (24). It consists of 12 items, each with five response options, scored on a scale from 0 to 4 (Daily, 2–3 times/week, Once/week, Occasionally, Never). The total score allows the identification of the level of eating habits, with higher scores indicating healthier dietary practices. Examples of the questions included in the questionnaire are: “How often do you consume vegetables?” and “How often do you consume fruits?.” The instrument showed acceptable reliability in its recent adaptation (*α* = 0.786).

### Educational program and intervention process

The nutritional educational program *“Small Steps Toward a Healthy Life”* was implemented over a two-month period—April and May 2025—at a public school in the district of Lurigancho-Chosica, Lima Metropolitan Area, Peru. Specifically, the sessions were conducted once per week for six consecutive weeks, while one preparatory week was allocated for logistical organization and the random selection of students, and one final week was dedicated to closing activities. In the intervention applied to the experimental group, a theoretical–practical approach was implemented, carefully adapted to the students’ age and school context. Dynamic and participatory activities were incorporated to facilitate content comprehension and to encourage the adoption of positive changes in daily eating habits. The intervention process comprised four phases. In the planning phase, the project was presented to teachers, parents, and students, the objectives were explained, and informed consent and assent were obtained. In the initial phase, the first nutritional assessment (pretest) was carried out using the eating habits questionnaire. The implementation phase consisted of six educational sessions delivered to adolescents and parents, distributed over the two-month intervention period, and complemented with practical activities to promote healthy eating habits. The sessions for adolescents included: (1) current panorama of child nutrition in Peru and prevention of NCDs, (2) healthy eating and essential nutrients, (3) food groups and frequency of consumption, (4) healthy vs. processed foods, (5) importance of water intake, and (6) a practical workshop on preparing healthy lunchboxes and meals. Additionally, two specific sessions were conducted for parents: the first on healthy eating and essential nutrients for child development, and the second, a practical healthy cooking workshop with demonstrations and participatory contests. In the final evaluation phase (posttest), the eating habits questionnaire was reapplied to assess the effectiveness of the nutritional educational program among adolescents. Additionally, two specific sessions were delivered to parents, reinforcing the intervention through family involvement.

### Statistical analysis

Statistical analyses were performed using SPSS version 29 and RStudio version 4.3.2. Descriptive statistics were applied, including measures of central tendency (M) and dispersion (SD). Data normality was assessed using the Shapiro–Wilk test. To compare eating habits scores before and after the intervention within each group, the Wilcoxon signed-rank test was used, whereas between-group comparisons were performed using the Mann–Whitney U test. Effect sizes were calculated using Cohen’s *d*. A statistical significance level of *p* < 0.05 was considered.

## Results

Table 2 presents the results of the comparison of eating habits before and after the implementation of the nutritional program. In the experimental group, mean scores increased significantly from 26.06 (SD = 1.69) at pretest to 30.88 (SD = 1.99) at posttest. The Wilcoxon signed-rank test indicated that this difference was statistically significant (*Z* = −3.542, *p* < 0.001). The effect size, calculated as Cohen’s *d* = 2.61 (95% CI: 1.58, 3.64), indicates a very large effect, demonstrating a substantial improvement in eating habits as a result of the intervention. In contrast, the control group showed a slight decrease in mean scores, from 26.00 (SD = 3.12) at pretest to 24.75 (SD = 5.28) at posttest. This difference was not statistically significant (*Z* = −0.682, *p* = 0.495), and the effect size was small [*d* = −0.29 (95% CI: −1.00, 0.42)], indicating that no meaningful improvements in eating habits occurred without the application of the program.

**Table 2:** Comparison of eating habits before and after the implementation of the nutritional program.

	Pretest		Posttest					
Group	M	SD	M	SD	Z^a^	*d^b^*	95% CI (*d*)	*p*
Experimental (*n* = 16)	26.06	1.69	30.88	1.99	−3.542	2.61	[1.58; 3.64]	<0.001
Control (*n* = 8)	26.00	3.12	24.75	5.28	−0.682	−0.29	[−1.00; 0.42]	0.495

Wilcoxon signed-rank test. Data did not follow a normal distribution according to the Shapiro–Wilk test (*p* < 0.05). ^a^Standardized Wilcoxon statistic. ^b^d = d de Cohen (Effect size). M, Mean; SD, Standard Deviation; CI, Confidence Interval. All significance tests were two-tailed.

Table 3 presents the comparison of eating habits between the experimental and control groups before and after the implementation of the nutritional program. At pretest, the mean scores were very similar between both groups (*M* = 26.06, SD = 1.69 in the experimental group; *M* = 26.00, SD = 3.12 in the control group). The Mann–Whitney U test indicated no statistically significant difference (*Z* = −0.094, *p* = 0.928), and the effect size, calculated using Cohen’s *d* = 0.003 (95% CI: −0.87, 0.93), confirmed the absence of an effect. At posttest, however, the results showed a clear divergence. The experimental group achieved a mean score of 30.88 (SD = 1.99), whereas the control group obtained a mean score of 24.75 (SD = 5.28).

**Table 3:** Comparison of eating habits between the experimental group (*n* = 16) and the control group (*n* = 8) before and after the nutritional intervention.

	Pretest (EG)		Pretest (CG)					
	M	DS	M	DS	Z^a^	*d^b^*	95% CI (*d*)	*p*
Eating habits	26.06	1.69	26.00	3.12	−0.094	0.003	[−0.87; 0.93]	0.928
	Posttest (EG)		Posttest (CG)					
Eating habits	30.88	1.99	24.75	5.28	−2.845	1.80	[0.75; 2.85]	0.004**

Mann–Whitney U test. Data did not follow a normal distribution according to the Shapiro–Wilk test (*p* < 0.05). ^a^Standardized Mann–Whitney U statistic. ^b^d = d de Cohen (Effect size). *p* statistically significant (** < 0.01). M, Mean; SD, Standard Deviation; CI, Confidence Interval. All significance tests were two-tailed. EG = Experimental Group; CG = Control Group.

The Mann–Whitney U test revealed a statistically significant difference (*Z* = −2.845, *p* = 0.004), with a large effect size [*d* = 1.80 (95% CI: 0.75, 2.85)], indicating a substantial impact of the intervention.

## Discussion

The results of this study demonstrated that the implementation of the brief nutritional educational program in the experimental group produced a statistically significant improvement in eating habits after the intervention (*p* < 0.001), whereas no relevant changes were observed in the control group (*p* = 0.495). When comparing both groups, no significant differences were found at the pretest phase (*p* = 0.928); however, at posttest a significant difference was observed in favor of the experimental group compared with the control group (*p* = 0.004). These findings confirm the effectiveness of the intervention, showing that participants in the experimental group achieved a substantial and statistically superior improvement compared to the control group.

These findings are consistent with previous evidence reporting improvements in knowledge, diet quality, and eating behaviors following school-based interventions, although many of those interventions were implemented over longer periods (e.g., 22 weeks or up to 2 years) (10–12). Specifically, our results align with short-term intervention programs that have demonstrated significant changes. For example, a brief school-based program in adolescents increased fruit and vegetable intake while reducing the consumption of soft drinks (16). Similarly, a 10-week intervention in children living under vulnerable conditions improved healthy habits related to fruit and vegetable consumption (14). Likewise, a short-term pilot program in primary schoolchildren reduced excess weight and promoted more favorable eating behaviors (13). In addition, another school-based nutritional program successfully decreased the intake of free sugars and improved nutritional knowledge among fifth-grade students (15). Similarly, a brief educational intervention in Croatian schoolchildren enhanced nutritional knowledge, diet quality, and lifestyle within only a few weeks (8). Therefore, the available evidence supports the effectiveness and feasibility of brief educational interventions, particularly when they are culturally relevant and implemented through dynamic methodologies.

A relevant factor that may have contributed to the effect observed in the intervention of our study could be related to an increase in the consumption of vegetables and fruits, as well as a reduction in the intake of junk food and sugar-sweetened beverages. This pattern of change is consistent with the findings reported by Ahmed et al. (16) and López et al. (14). For instance, Ahmed et al. (16), after implementing their school-based program in adolescents, observed an increase in fruit and vegetable consumption and a decrease in soft drink intake. This trend could be explained by the fact that the contents of such programs emphasize the importance of increasing the consumption of fresh and natural foods and reducing the intake of ultra-processed products, which aligns with the most common approaches in school-based interventions aimed at promoting healthy eating. The relevance of this finding lies in the fact that changes in specific dietary components—such as a higher intake of fruits and vegetables and a lower consumption of sugar-sweetened beverages—are considered key indicators of diet quality and are associated with a reduced risk of obesity, type 2 diabetes, and cardiovascular diseases (25–27).

### Implications for public health

The findings of this study provide preliminary indications that brief school-based nutritional education interventions involving parental participation could be a promising approach to encouraging healthy eating habits among adolescents. While further evidence is required, their incorporation into public health programs could potentially contribute to preventing the early adoption of unhealthy dietary behaviors associated with overweight, obesity, and non-communicable diseases (NCDs). Moreover, these preliminary results indicate that such programs may be feasible to replicate in public schools across different contexts in Peru, which could open the possibility of contributing to the reduction of nutritional health inequalities and to strengthening food and nutrition education as a key component of preventive policies. Taken together, these findings not only support the potential of short-term interventions as preventive tools in public health, but also challenge the notion that only long-term approaches are capable of producing meaningful change. In fact, the results suggest that well-structured and culturally adapted brief programs may generate initial improvements in adolescents’ eating habits. In contexts such as Peru—characterized by the coexistence of excess weight and socioeconomic vulnerability—this type of intervention could serve as a preliminary step toward more comprehensive strategies that integrate curricular, family, and school-environment components aimed at sustaining behavioral change over time.

### Limitations and recommendations for future research

This study presents some limitations that should be considered when interpreting the results. First, the relatively short intervention period does not allow conclusions about the sustainability of the observed changes over the long term. Another limitation is the reliance on a self-reported questionnaire, which may be subject to memory bias or social desirability bias among students. In addition, the quasi-experimental design limits the ability to draw strong causal inferences, as it does not control for potential confounding variables with the same level of rigor as a randomized controlled trial. Regarding recommendations for future research, efforts should focus on strengthening the comprehensive approach of nutritional interventions. In this sense, it is suggested to include the evaluation of physical activity, as it is an essential component of a healthy lifestyle and directly influences the overall health of adolescents. It is also advisable to incorporate anthropometric indicators such as body mass index (BMI), waist circumference, or skinfold thickness in order to more comprehensively assess the nutritional effects of the intervention. Moreover, fostering the active involvement of families through awareness strategies and direct participation is crucial—particularly in contexts where caregivers have low educational levels—to enhance the impact of interventions on adolescents’ eating habits. Similarly, it is recommended to employ instruments with stronger evidence of validity and reliability, or alternatively, to use 24-h dietary recalls to obtain a more detailed quantification of nutritional habits. Finally, in terms of generalizability, although the sample size was small, the consistency between the large within-group effect and the significant between-group difference suggests a robust result for the context of the intervention. To advance toward stronger and more transferable inferences, it would be pertinent to replicate the intervention with larger samples, include medium- and long-term follow-ups (e.g., at 3, 6, and 12 months), and align the educational component with environmental changes (e.g., school kiosk regulations, availability of safe drinking water, front-of-package labeling, and protected time for healthy snacks, among others).

## Conclusion

The findings of this study provide preliminary indications that the implementation of a brief nutritional education program may contribute to improvements in eating habits among adolescents in the experimental group, compared to the stability observed in the control group. Although both groups exhibited equivalent conditions at baseline, the improvements observed in the experimental group should be interpreted with caution, considering the limitations of the study (small sample size, reliance on self-report, short intervention duration, and lack of follow-up). Accordingly, the results should not be regarded as conclusive evidence of effectiveness, but rather as initial indicators of feasibility and short-term impact, which support the potential applicability of brief educational interventions in school settings. Furthermore, these findings suggest that low-cost and easily implementable programs could serve as complementary strategies for health promotion in public and private schools, provided that they are replicated and evaluated in future studies with larger samples and longitudinal follow-up. Finally, we recommend continuing this line of research through larger-scale controlled studies that may confirm the observed effects and determine their sustainability over the medium and long term.

## Data Availability

The raw data supporting the conclusions of this article will be made available by the authors, without undue reservation.
